# Notes and News

**Published:** 1902-05-03

**Authors:** 


### Notes and News.

Five thousand pounds have been promised to the Cancer Research Fund provided other like sums are forthcoming.

A new operating theatre has been added to the Victoria Burnley Hospital. It is the gift of Mr. John Witham, is completely equipped, and cost about £1,000.

Lord Monkswell will preside at the festival dinner of the Newsvendors' Benevolent and Provident Institution on Wednesday, May 7th, at the Trocadero Restaurant.

A new isolation hospital has been opened for the Fylde District Union. The hospital comprises pavilions for scarlet fever, typhoid, and diphtheria, administrative buildings, and the usual offices.

A wing of the Newcastle Infectious Diseases Hospital has been opened by Mr. Stainer and presented to the Corporation, in accordance with the wishes of his father, the late Mr. Francis Stainer. after whom it is named. The wards are in one-storeyed detached pavilions, and contain from two to 12 beds in each.

In the new forms of death certificates issued to medical practitioners, the duty of the practitioner in regard to the certification of cases of death from violence is definitely laid down. Thus among the "Suggestions" the following appears: "12. Violence.—In every case of death by Violence, or by suspected Violence, the Medical Practitioner, in addition to stating the fact in his certificate, should advise the friends of the deceased to inform the Coroner forthwith." Thus, notwithstanding the statements of various coroners to the contrary, it is the duty of the practitioner, even in cases of violence, to give a certificate. All he has to do is to take care that it states the fact, or the suspicion, of violence, and that he advises the friends of the deceased to inform the coroner. There is no suggestion that the practitioner should himself inform the coroner, or that he should break the law, as he has sometimes been advised to do, by refusing the certificate.

Scarlet fever has lately been more prevalent and fatal in Chicago than during the past seventeen years.

The number of plague cases in the Punjaub continues very large. There are now no cases in Mauritius.

The last Swedish census recorded the lowest death-rate ever yet attained by a civilised nation. During the last decade it only reached 16·49 per 1,000 persons.

The London County Council has given notice that chicken-pox will remain a notifiable disease for a further period of six months, from May 6th, throughout the administrative county of London.

A Sanitary Conference was held at Manchester this week, attended by delegates of societies interested in sanitary work, to celebrate the jubilee of the Manchester and Salford Sanitary Association.

A new Infectious Diseases Hospital has been opened for Bromsgrove, Droitwich, and Redditch. It succeeds a temporary structure. The hospital consists of blocks placed round a square, the wards containing varying numbers of beds. The cost of the buildings and their equipment is upwards of £13,000.

A large ball is to be held at the Crystal Palace on July 2nd to augment the Coronation Gift which is to be devoted to King Edward's Hospital Fund for London. The honorary secretaries are Lady Maud Wilbraham, Lady Raymond West, and Mrs. E. Colgrave. All particulars will be supplied by the secretary, 20 Victoria Street, S.W

A petition is to be presented by the Liverpool City Council to the King in Council praying for a Charter for the Foundation of a University in Liverpool. Leeds is opposing any attempt to divide the Victoria University. Nearly £157,000 has already been promised towards the foundation of the new University, and the feeling locally is very strong that Liverpool is important enough to have its own University.

Dr. Roddick's Bill for Dominion Medical Registration has been twice read in the Dominion House of Commons. It has been reported upon favourably by a Special Committee of the House, and it is considered probable that the Bill will pass. The Bill is only permissive, it does not take away any existing rights of Provinces, and it does not compel any of them to take part in the formation of the Dominion Medical Council. Before the measure becomes absolute, however, it is necessary that all Provincial Legislatures shall ratify it.

Although we still hold much aloof in England from the introduction of the paying patient, our colonies are becoming alive to the necessity and advantage of the system. At the Hôtel Dieu Hospital, in Montreal, a new wing is about to be added consisting mainly of private wards, as the demands for these are found to increase. At the same hospital a new operating room and special room for X-ray apparatus will be provided, to increase the facilities offered to the students of the Laval University.

